# Creation and implementation of an emergency medicine education and training program in Turkey: an effective educational intervention to address the practitioner gap

**DOI:** 10.1186/1865-1380-6-29

**Published:** 2013-07-22

**Authors:** Jennifer Whitfield Bellows, Katherine Douglass, Ridvan Atilla, Jeffrey Smith, G Bobby Kapur

**Affiliations:** 1 Department of Emergency Medicine Denver Health Medical Center, University of Colorado School of Medicine, 777 Bannock Street Mail Code 0108, Denver, Colorado 80204, USA; 2 Department of Emergency Medicine, The George Washington University, 2150 Penn. Ave. Suite 2B-417, Washington, DC 20037, USA; 3 Department of Emergency Medicine, DokuzEylul University Hospital, Balcova, 35340, Izmir, Turkey; 4 Section of Emergency Medicine Baylor College of Medicine Emergency Center, 1504 Taub Loop, Houston, TX 77030, USA

**Keywords:** “Train-the-trainers,” Turkey, International emergency medicine, Disaster medicine, Education

## Abstract

**Background:**

The specialty of Emergency Medicine has enjoyed recognition for nearly 20 years in Turkey. However, the majority of underserved and rural Turkish emergency departments are staffed by general practitioners who lack formal training in the specialty and have few opportunities to increase emergency medicine-specific knowledge and skills.

**Methods:**

To address this “practitioner gap,” the authors developed a four-phase comprehensive emergency medicine education and training program for general practitioners practicing in government hospitals in Turkey.

**Results:**

From April 2006 until June 2009, 42 courses were taught by 62 trainers across seven regions in Turkey. A total of 2,262 physicians were trained. The mean course pre-test score for all regions was 42.3 (95% CI 39.8 to 44.7). The mean course post-test score was 70.1 (95% CI 67.2 to 72.9). The difference between the mean scores was 27.8 (95% CI 25.3 to 30.4, *P* <0.0001), reflecting an improvement of 65.7%.

**Conclusions:**

A partnership between an academic emergency medicine department and an emergency medicine society to implement country-wide training of physicians practicing in public emergency departments can serve as a successful model for capacity-building global emergency medicine endeavors.

## Background

Emergency medicine (EM) as an independent medical specialty continues to spread actively throughout the world. Over the last 20 years, medical communities in a growing number of countries have recognized EM as a specialty and established training programs to train new EM physicians [1]. Specifically in Turkey, EM was recognized as a specialty in 1993, at which time the Emergency Medicine Association of Turkey (EMAT) was founded. Though many medical specialties in Turkey enjoyed highly developed systems and technology by the early 1990s, pre-hospital care and emergency medicine were at that time described as “in their infancy” [2].

Since then, the specialty has experienced rapid growth and development. The country’s first emergency medicine residency was established in 1994 in Izmir [3]. Recent pressure from the Ministry of Health to provide EM residency training in public hospitals has precipitated rapid growth of residencies; now there are 80 such programs throughout Turkey [4,5]. However, only 23 of these programs have graduated residents as the training period is 5 years and many of these programs are very new. Increasing academic productivity has led to the creation of two Turkish emergency medicine journals, and the country has been host to several international EM conferences [5].

Despite this significant interest and dedication to the growth of EM as a specialty, a significant shortage of EM-trained physicians remains in Turkey, particularly in rural and underserved areas. This ‘Practitioner Gap’ is a phenomenon common to nascent Emergency Medicine systems: as new training opportunities arise for those going through residency programs, emergency departments in most areas are still staffed by general practitioners with little or no formal specialized EM training. These practitioners are not required to pursue continuing medical education, and access to new information, education, and further training is very limited. As of September 2009, there were approximately 460 EM residency trained physicians practicing in Turkey and approximately 10,000 general practitioners working in emergency departments (EDs) throughout the country [5].

## Methods

In an effort to address this education and training gap, The Ronald Reagan Institute of Emergency Medicine (RRIEM) at the George Washington University (GWU) in cooperation with EMAT conducted a multi-phase modular training program on the management of critical issues in emergency medicine in Turkey. Cooperative discussion between RRIEM/GWU and EMAT in planning the project identified the most significant need for the EM system in Turkey to be training for these ‘untrained’ practitioners working in emergency departments. Historical data and clinical needs led to the decision that laying a foundation of basic EM skills, including triage, rapid assessment and prioritization of emergency interventions, and general knowledge of EM management, was the most effective method to prepare general practitioners to respond to both daily emergency care and occasional disaster events. The aim of the project was to create a uniform and consistent knowledge and skill set among general practitioners working in Turkish emergency departments. The project was financially supported by Americares, a US-based non-profit international relief organization, and in-kind support from the faculty of the RRIEM/GWU.

The stated goal of the project was to enhance coordination and interactions among stakeholders within Turkey in the areas of emergency medicine and disaster response and to improve the delivery of emergency medical care through a comprehensive training program for general practitioners working in emergency departments. In order to achieve this, the project was divided into four phases (Table 1).

**Table 1:** Timeline and summary of the emergency medicine and disaster preparedness project, Turkey, 2006-2009

**Phases**	**Title**	**Duration**	**Description**
**Phase I**	Disaster preparedness and response	March-June 2006	Preparation for and implementation of a 1-day course on disaster preparedness held in capital city of Ankara
**Phase II**	Stakeholders conference and preparation	July 2006	Two-day meeting on state of emergency medicine both internationally and in Turkey, EM program development, leadership, and pre-hospital care systems, with the goal of forming consensus for goals and objectives of project
**Phases III**	Train-the-trainers conference	Nov 2006	Five-day conference on core EM topics given collaboratively by faculty from EMAT and GWUMC to the regional coordinators. This was followed by final organization and translation of conference materials into a 4-day EM course, development of a web-based site containing all course materials, logistics planning for course administration, and production of class handouts
**Phase IV**	Course implementation	Nov 2006-May 2009	Administration of the 4-day EM course to Turkish EM practitioners in 7 regions of Turkey

Phase I occurred from March through June 2006 and focused on disaster preparedness. The RRIEM/GWU team created a 1-day Disaster Preparedness and Response Course that was held in the Turkish capital of Ankara. The course consisted of case-based lectures and a disaster drill that simulated a double-bombing incident in the city. The course itself was held on 30 June 2006.

Phase II consisted of a Stakeholders’ Meeting. It included the principal directors from RRIEM/GWU and EMAT; members of the Turkish, European, and Asian emergency medicine community, including university faculty; general practitioners; representatives from physician and non-physician pre-hospital Emergency Medical Systems (EMS) groups; and government officials. There were also lectures devoted specifically to pre-hospital care, academic emergency medicine program development, and residency training. The conference format consisted of lectures as well as small group discussions and question-and-answer sessions.

**Figure 1:**
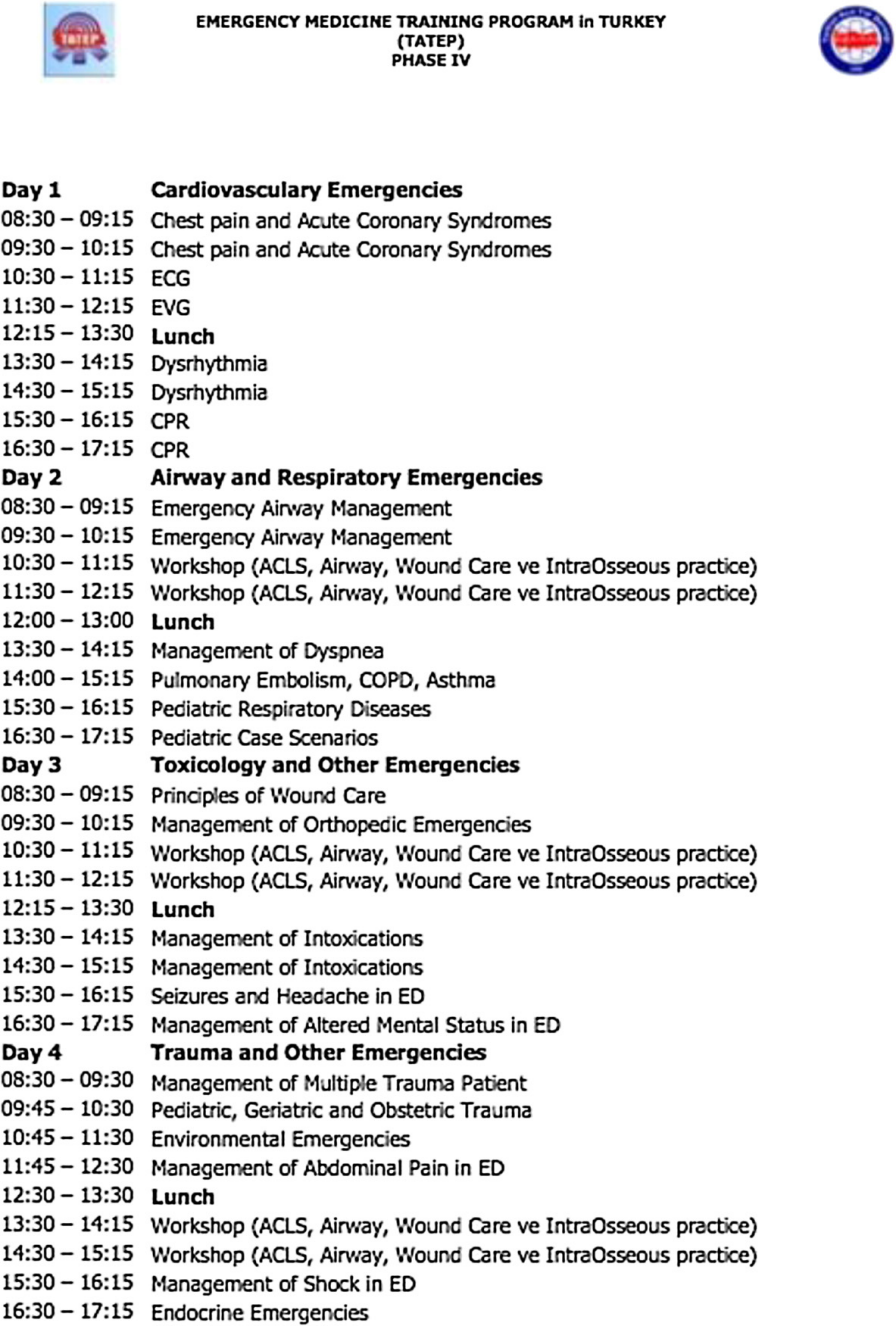
Four-day core topics in emergency medicine: course curriculum.

Phase III of the project was the collaborative development of the EM course. Project developers at RRIEM/GWU and EMAT created the course curriculum, materials, and evaluations that reflected the most relevant topics for Turkish physicians in government hospital emergency departments. The resulting course curriculum is detailed in Figure 1.

The ‘Training-of-Trainers’ course allowed time for discussion among participants regarding content and logistics of course implementation. Separate educational sessions were delivered on the topic of effective educational strategies. The final consensus curriculum was a 4-day course to allow administration over two weekends as necessary. Lectures were converted to a text format to make a “textbook” of the lectures that was then translated into Turkish and given to the trainers and students. Pre-test, post-tests, and course evaluations were also created and included in course materials to allow for feedback and ongoing evaluation of the courses. A website was launched that contained the teaching materials for the instructors.

Phase IV was the most critical project phase: course administration and evaluation. The trainers taught the courses in seven regions in Turkey from 2006–2009. The first 4-day course was taught in Izmir, Turkey, in April 2007. The participants were given pre-tests and post-tests during each course and were asked to complete course evaluations. In addition, focus groups were convened to gather qualitative data.

The Phase I Disaster Preparedness Course had 48 participants, and the Disaster Drill allowed the participants to focus on a specific event for Turkey. The participants were brought together from across the country, and it gave them an opportunity to share ideas and concepts that were being implemented in different regions.

The Phase II Stakeholders’ Meeting included approximately 70 participants from a diverse scope of clinical practitioners and government officials. In addition, EM leaders from other countries provided contextual input. Stakeholders at the 2-day meeting discussed the state of emergency medicine in different regions of the country, evaluated current practice standards, and identified clinical needs, training strategies, and possibilities for a common curriculum for future EM training. One of the primary outcomes of the meeting was developing a shared and united consensus for increased training and education for practitioners in emergency departments in government hospitals in Turkey.

**Figure 2:**
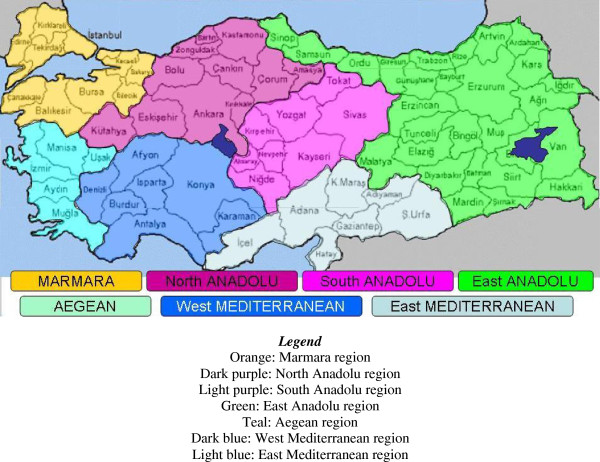
Seven regions in Turkey with primary cities.

For the Phase III Curriculum Development and Train-the-Trainers Course, 62 emergency physicians representing seven designated regions of Turkey were chosen as trainers and invited to a 5-day “Train-the Trainers” course held in November 2006 in Antalya, Turkey. Trainers were selected by EMAT and RRIEM/GWU collectively, based on suitability as well as population density. Quite simply, more trainers were supplied in more populated regions. A lead center, associated with site trainers, was established for each region. Five of seven regions had lead centers that were university-based or affiliated with established EM programs. The remaining two regions, located in the eastern provinces, were supervised by EM physicians affiliated with large, non-academic hospitals. A map of the seven regions and their major cites is provided in Figure 2.

Phase IV Provider Training occurred from April 2007 until June 2009, and 42 courses were administered across the seven regions of Turkey. A total of 2,262 physicians were trained through these courses. To analyze the efficacy and general reception of the course, several evaluative methods were employed. Tests of general emergency medicine knowledge (“pre-tests” and post-tests”) were administered before and after the course for each participant.

## Results

The mean pre-test score for all regions was 42.3 (95% CI 39.8 to 44.7). The mean post-test score was 70.1 (95% CI 67.2 to 72.9). The difference between the mean scores was 27.8 (95% CI 25.3 to 30.4, *P* <0.0001), reflecting an improvement of 65.7%.

Additionally, course evaluations were distributed to the participants several months after the course. Finally, focus groups were held periodically in September 2007, October 2008, and May 2009 between the GWU stakeholders, EMAT directors, course trainers, and participants to obtain dynamic feedback.

Additional feedback and evaluation regarding the course were gained on a continual basis via communication among stakeholders as well as several focus group interviews with both instructors and participants. This ongoing evaluative process allowed stakeholders to identify course-related challenges and to create solutions to those issues. Major themes that were identified included: variability among practice environments for practitioners and related challenges associated with course content, the range of baseline knowledge of course participants, curriculum challenges in terms of content and methodology, as well as logistical issues with course delivery such as timing of courses and availability of materials.

## Discussion

The practice settings and style for the general practitioners are different from those encountered in the US; often the general practitioners are seeing up to 500 patients a day. This results in a relatively higher reliance on triage and transfer skills with less usage of ancillary tests, especially CT and ultrasound. Additionally, according to some participants, informal mandates from the Turkish Ministry of Health encourage general practitioners to limit the scope of their interventions for critically ill patients, encouraging them to concentrate on rapid cursory stabilization and immediate transfer to tertiary care facilities and/or the care of consultants. Reliance on transfer and consultant input varied widely by institution and region; participants at sites with emergency medicine training programs and recognized departments of emergency medicine did not follow this mandate as closely as those practicing in more remote, resource-poor environments. This reality manifested in a discrepancy in some settings between what was being taught in the courses and how some practitioners felt they were encouraged to practice.

Not surprisingly, the level of experience and expertise among participants varied as widely as practice style. For example, participants enrolled in residency training programs felt the course was helpful but not too difficult and did not identify lectures or topics as being too complex. Several general practitioners, however, found the lectures more challenging. Instructor feedback also revealed that some course participants lacked basic skills such as electrocardiogram interpretation and basic resuscitation. Despite these variances in practice style and experience, focus group participants uniformly felt the course increased their confidence and knowledge of proper clinical management, regardless of how well they could apply it to their practice in the emergency department.

Specifics regarding the course curriculum were raised as well. For example, detailed instruction in some topics such as endocrinology, neurology, and environmental medicine were perceived as less relevant to general practice than cardiopulmonary and trauma care. Also, physiology and theory instruction in general were seen as less important than practical teaching of diagnosis and management. Finally, and perhaps most uniformly among participants, was the desire to have more case-based and simulation-based learning as compared to traditional lecture style classes, as well as the desire for further instruction on procedures, cardiovascular emergencies, and trauma. A portion of the course was devoted to mock code simulations and procedural practice on mannequins. Participants repeatedly lauded these sessions as the most valuable and relevant to their practice, and they recommended further use of the mannequins in particular and more case-based learning in general in future courses. In response to this feedback, course instructors were given freedom to alter the curriculum to meet these requests. Future curricula will be modified as well in accordance with this feedback.

Occasional issues with the course logistics were also revealed. In some locations, particularly in Istanbul, the course was administered over 4 days in a row, while others were held over two sequential weekends. Unfortunately, it was difficult for some practitioners to attend a 4-day program because of challenges with shift coverage. As a result, future courses may be shortened to 3 days and/or be held over a series of evenings to allow scheduling flexibility. Transportation to and parking at course sites were sometimes difficult. Occasionally, course materials such as chicken legs to practice intra-osseous access were not available. In response to these logistical needs, EMAT and course instructors partnered with local pharmaceutical companies and the Turkish Ministry of Health to help transport participants to the course sites, provide lunch and needed supplies, and allow courses to be conducted at various locations.

For the final evaluation component of the project, stakeholders had a very low response rate in obtaining completed answers to the 3-month post-training questionnaire. The questionnaires were initially e-mailed as an attachment to the participants, but very few responded. It is likely these e-mails were filtered as “spam” or the e-mail address was not correct or current. To circumvent this issue, pharmaceutical representatives were again employed to deliver and collect the questionnaires personally from participants at their homes or hospitals. Through this method over 400 completed questionnaires were made available for review and are now in the process of being analyzed.

### Cost effectiveness

The total budget for implementation of this project was $545,300. With 2,262 persons trained, the cost per participant can be simply divided into $241 (USD) per person. This does not take into account the cost of curriculum development, project implementation, or evaluation. Further research needs to be carried out to determine the degree to which this improved knowledge brings about a change in morbidity, mortality, hospital flow, or other similar metrics. Further research also could explore the additional outcomes of the project including increased awareness of emergency medicine, improved collaboration and communication among key stakeholders, and improved disaster preparedness.

## Conclusions

The Emergency Medicine Training Program in Turkey was implemented to improve the uniform and consistent knowledge and skill set among general practitioners working in Turkish emergency departments, many of whom had previously received little or no formal emergency medicine training. This project demonstrates an effective methodology for widespread dissemination of emergency medicine knowledge. Many practitioners took the course and gained knowledge and access to information. The partnership between RRIEM/GWU and EMAT and the train-the-trainers format allowed the extensive distribution of the course to every region in Turkey. Focus group feedback was generally very positive; interviewed participants were happy they had taken the course. Ongoing feedback as well as creative problem solving by stakeholders and instructors alike allowed for modifications of the project as it was implemented and identification of issues that can be addressed in future courses. With limited formal training programs and very few qualified instructors at those programs, the ‘practitioner gap’ in emergency medicine remains a very significant issue in many countries. Nationally based training programs such as this can help increase the level of emergency medicine in a unique, effective, and efficient manner. As such, this project can serve as a model for similar future endeavors internationally.

## Abbreviations

EM: Emergency medicine; EMAT: Emergency Medicine Association of Turkey; GWU: George Washington University; RRIEM: The Ronald Reagan Institute of Emergency Medicine.

## Competing interests

The authors have no competing interests.

## Authors’ contributions

JWB was involved in implementation of the project and analysis of the test results, and drafted the manuscript. KD was a creator of the project, implemented and tracked its progress, and helped draft the manuscript. RA helped implement the project and helped draft the manuscript. JS was a creator of the project and helped draft the manuscript. GBK was a creator of the project, implemented and tracked its progress, and helped draft the manuscript. All authors read and approved the final manuscript.

## Authors’ information

Jennifer Whitfield Bellows, MD, MPH, is an assistant professor at the University of Colorado School of Medicine in the Department of Emergency Medicine at Denver Health Medical Center. She has subspecialty training in International Emergency Medicine.

Kate Douglass, MD, MPH, is an assistant professor at the George Washington University Department of Emergency Medicine. She is currently the International Emergency Medicine and Global Health Fellowship Director at GWU.

Ridvan Atilla, MD, MPH, is an assistant professor at the University of Dokuz Eylul School of Medicine in the Department of Emergency Medicine at Izmir, Turkey. He was one of the founding members of the Emergency Medicine Association of Turkey (EMAT) in 1995 and the president of EMAT between 2007–2009. His professional interests are cardiac emergencies, trauma management, emergency ultrasound and disaster medicine.

Jeffrey Smith, MD, MPH, is an associate professor and chief of the International Emergency Medicine Section at the George Washington University Department of Emergency Medicine. He is also co-director of the Ronald Reagan Institute of Emergency Medicine in Washington, DC.

G. Bobby Kapur, MD, MPH, is the Associate Chief for Academic Affairs and Residency Program Director at Baylor College of Medicine and Associate Chief of the Emergency Center at Ben Taub General Hospital in Houston, TX.
